# Detection of minimal residual disease in acute myeloid leukemia: evaluating utility and challenges

**DOI:** 10.3389/fimmu.2024.1252258

**Published:** 2024-06-13

**Authors:** Noemí Álvarez, Alejandro Martín, Sara Dorado, Rafael Colmenares, Laura Rufián, Margarita Rodríguez, Alicia Giménez, Laura Carneros, Ricardo Sanchez, Gonzalo Carreño, Inmaculada Rapado, Yanira Heredia, Joaquín Martínez-López, Santiago Barrio, Rosa Ayala

**Affiliations:** ^1^ Hematology Department, Hospital Universitario 12 de Octubre, Instituto de Investigación Sanitaria Imas12, Madrid, Spain; ^2^ Hematological Malignancies Clinical Research Unit, Centro Nacional de Investigaciones Oncológicas (CNIO), Madrid, Spain; ^3^ Altum Sequencing Co., Madrid, Spain; ^4^ Computational Science Department, Carlos III University, Madrid, Spain; ^5^ Department of Medicine, Complutense University of Madrid, Madrid, Spain; ^6^ Centro de Investigación Biomédica en Red de Cáncer (CIBERONC), Instituto Carlos III, Madrid, Spain

**Keywords:** AML, liquid biopsy, MRD, NGS, multiparametric flow cytometry, Leukaemia

## Abstract

This study discusses the importance of minimal residual disease (MRD) detection in acute myeloid leukemia (AML) patients using liquid biopsy and next-generation sequencing (NGS). AML prognosis is based on various factors, including genetic alterations. NGS has revealed the molecular complexity of AML and helped refine risk stratification and personalized therapies. The long-term survival rates for AML patients are low, and MRD assessment is crucial in predicting prognosis. Currently, the most common methods for MRD detection are flow cytometry and quantitative PCR, but NGS is being incorporated into clinical practice due to its ability to detect genomic aberrations in the majority of AML patients. Typically, bone marrow samples are used for MRD assessment, but using peripheral blood samples or liquid biopsies would be less invasive. Leukemia originates in the bone marrow, along with the cfDNA obtained from peripheral blood. This study aimed to assess the utility of cell-free DNA (cfDNA) from peripheral blood samples for MRD detection in AML patients. A cohort of 20 AML patients was analyzed using NGS, and a correlation between MRD assessment by cfDNA and circulating tumor cells (CTCs) in paired samples was observed. Furthermore, a higher tumor signal was detected in cfDNA compared to CTCs, indicating greater sensitivity. Challenges for the application of liquid biopsy in MRD assessment were discussed, including the selection of appropriate markers and the sensitivity of certain markers. This study emphasizes the potential of liquid biopsy using cfDNA for MRD detection in AML patients and highlights the need for further research in this area.

## Introduction

The prognosis of newly diagnosed acute myeloid leukemia (AML) patients is based on biological criteria such as age, number of leukocytes in the peripheral blood, and cytogenetic and molecular alterations. NGS has revealed the heterogeneous molecular complexity of the AML genome, especially in cytogenetically normal acute myeloid leukemia (CN-AML) (1–3), which has contributed to the refinement of risk stratification (4–6) and personalized therapeutic strategies for these patients (7, 8).

However, long-term survival is less than 30% in patients below the age of 60 years and worse in older AML patients or with comorbidities (9, 10). Additionally, a significant percentage of patients with intermediate risk and even good prognosis will experience treatment failure, usually in the form of leukemia relapse and less frequently refractoriness, even after initially achieving complete remission post-induction chemotherapy (11). Thus, after treatment, the presence of minimal residual disease (MRD) in patients who have achieved complete remission is a significant independent prognostic indicator in AML (12). Therefore, the quantification of MRD is vital for defining the best strategy for response consolidation, assessing the need for intensification treatment before consolidation, and providing early therapeutic interventions at relapse.

At present, the most widely used methods for the detection of MRD are multiparametric flow cytometry (MFC) and quantitative polymerase chain reaction (qPCR), applicable to around 90% and 50% of patients, respectively (12). Flow cytometry offers the advantage of high sensitivity, allowing the detection of aberrant immunophenotypic profiles characteristic of leukemic cells. However, this technique has less specificity, and its analysis is more subjective than other MRD techniques (13). QPCR enables the detection of fusion transcripts or specific leukemia mutations, providing high sensitivity and specificity. This method is particularly valuable in AML subtypes characterized by recurrent cytogenetic abnormalities. Nevertheless, qPCR is limited to detecting known mutations or fusion transcripts and may not capture the full spectrum of genetic alterations present in AML, especially in cases with complex or heterogeneous mutational profiles (14).

However, new sequencing methods to detect genomic aberrations present in about 98% of AML patients for use as MRD biomarkers are also being incorporated into clinical practice (4, 15). In general, all these studies use bone marrow samples as the starting material. Although there is an increasing number of studies comparing BM and PB samples, with the latter showing earlier detection of relapse in both adult AML and pediatric cases (16, 17).

The quantification of MRD is only performed in cases with mutated *NPM1* or CBF-positive AML CBF by targeting peripheral blood leukocyte DNA, and these data have been incorporated into clinical protocols (e.g., Pethema) or following the ELN-2022 recommendations. In other AML subtypes, once patients attain clinical remission, they undergo repeat bone marrow (BM) aspiration every 2 to 3 months during the first year and every 3 to 6 months for the next two years after the end of consolidation chemotherapy (6, 18). This involves performing numerous BM puncture aspirations, an uncomfortable and inconvenient procedure that can be avoided by using other MRD approximations such as liquid biopsy. In addition, the study of BM entails other limitations (19) which can be overcome using cfDNA, such as the fact that a single bone marrow specimen represents only a very small fraction of the total bone marrow cellular population (18); the spatial heterogeneity of leukemia (20); and the occurrence of extramedullary disease (21, 22).

Nakamura (23) and Short (24) studied the impact of cfDNA on leukemia relapse prediction in AML by using NGS to identify the driver mutation at diagnosis. Then tracking it using droplet digital polymerase chain reaction (ddPCR) (in Nakamura’s case, with 53 patients who were consolidated with allogeneic transplantation) or targeted NGS (in Short’s case, with 22 patients) for MRD quantification.

However, the utility of cfDNA in the detection of genomic aberrations related to tumor persistence in AML and its advantage over other specimens studied has not yet been demonstrated.

## Methods

### Patient cohort and samples

This study was performed in a cohort of 24 patients diagnosed with AML between 2019 and 2021 at the Hospital 12 de Octubre, Madrid. The median age at diagnosis was 58.5 (range 41–84) years. Fit patients received 3 + 7 or similar treatment schemes (i.e., patients under 65 years of age who were candidates for intensive chemotherapy and transplantation) (n=11), and venetoclax-, azacitidine-, or decitabine-based treatment for unfit patients (patients over 65 or with high comorbidities) (n=9). The study was conducted in accordance with Spanish Law 14/2007 on biomedical research and was approved by the Ethics Committee of the Hospital Universitario 12 de Octubre. All patients provided informed consent. The main characteristics of the patients are summarized in **Table 1** and the clinical-biological characteristics are shown in **Supplementary Table 1**.

**Table 1:** Clinical description of patients.

Patients (n = 20)	
Sex	
Male	6 (30%)
Female	14 (70%)
Age at diagnosis, median (range)	58.5 (41–84)
Blasts at diagnosis, median (range)	
Bone marrow samples	27.5 (9–80)
Peripheral blood samples	19.5 (1–85)
Leukocytes at diagnosis, median (range) 10^9/L	2.85 (0.1–8.6)
Death	
No	13 (65%)
Yes	7 (35%)
Relapse	
No	12 (60%)
Yes	8 (40%)
Hematopoietic stem cell transplantation	
No	5 (25%)
Allogeneic	13 (65%)
Autologous	2 (10%)
ELN2022 classification	
Favorable	6 (30%)
Intermediate	3 (15%)
Adverse	11 (55%)
Type of treatment	
Intensive	18 (90%)
Non-intensive	2 (10%)

A total of 74 follow-up samples obtained from peripheral blood (PB) and stored at -80°C prior to use were analyzed. These samples were collected post-induction and post-consolidation. Circulating tumor cells (CTCs) (n=39) and cell-free DNA (cfDNA) (n=35) were studied. 10 ml of PB were obtained and collected in streck tubes to obtain cfDNA and in EDTA tubes for the study of CTCs. For 24 patients, both fractions were available. We focused on these samples in our analyses. In addition, for 20 patients, we compared our results with those obtained with multiparametric flow cytometry (MFC).

A median of 28.26 ng of cfDNA and 1038.75 ng of genomic DNA (gDNA) was obtained for analysis. The minimum amounts of DNA required for sequencing were 15 ng for cfDNA samples and 660 ng for gDNA samples. All samples that did not reach the minimum amount were excluded from further analysis (In the case of the cfDNA samples 5 did not reach the minimum number, and in the case of the CTCs samples there were 7).

The cfDNA was obtained using the QIAamp Circulating Nucleic Acid Kit (ref. 55114, Qiagen) following the manufacturer’s instructions, after obtaining the plasma by centrifugation.

The Maxwell^®^ 16 Blood DNA Purification Kit (Promega Biotech Iberica, SL) is used to select the CTCs according to the manufacturer’s instructions.

### Mutational profile workflow

The mutational profile screening at diagnosis was defined by NGS (Ion Torrent System) using a custom panel of 42 genes frequently mutated in myeloid pathology (*ASXL1, BCOR, BCORL1, CALR, CBL, CSF3R, CEBPA, DNMT3A, EPAS1, EPOR, ETV6, EZH2, FLT3, IDH1, IDH2, JAK2, KDM6A, KIT, KMT2A, KRAS, MPL, NF1, NPM1, NRAS, PHF6, PRPF40B, RAD21, RUNX1, SETBP1, SF3A1, SF3B1, SH2B3, SMC1A, SRSF2, STAG2, TET2, THPO, TP53, U2AF1, VHL, WT1*, and *ZRSR2*). Only coding and splicing regions were included in this panel.

The workflow for filtering and classification of variants was performed as previously published by VAF and VAF kinetics in complete remission (25, 26).Somatic mutations were selected and used for MRD monitoring on liquid biopsy follow-up samples. MRD quantification was performed in the two main fractions, whole blood cells (WBC) and cell-free DNA (cfDNA), as previously mentioned (25). The threshold for MRD positivity was set at 10–^4^ (LiqBio-MRD test).

After the selection of somatic mutations as biomarkers of MRD, these mutations were amplified using PCR in triplicate with molecular-tagged primers (P1, P2, and P3). The PCR products obtained were combined in a single tube and the preparation of the libraries was continued. The final libraries were sequenced on the Ion S5 System platform (Life Technologies, Thermo Fisher Scientific Inc.) with 500,000x coverage per amplicon (25). Once the results were obtained, all triplicates with values of mean + 1 standard deviation were excluded from the analyses. In addition, the same pipeline was applied to three healthy control donor DNA samples to quantify the limit of detection (LOD) for each biomarker. The LOD was defined as the nine data points’ mean + 3 standard deviations. Therefore, if the VAF was lower than the LOD, the marker was excluded, and the MRD value was defined by the marker with the highest VAF in the sample (27).

### Bioinformatics and statistical analysis

The FASTQ files generated after sequencing were demultiplexed to separate reads from triplicates and amplicons. A rigorous bioinformatics process was carried out in Python (3.7.12 version) and R (3.6.1. version) to eliminate low-quality reads and reduce the level of false positives. Wild-type and mutated sequences were obtained for each of the gene positions. At least 15bp on each side needed to match exactly with the reference (WT) sequence. Then, the amplicons were generated to sizes of up to 140bp, aiming to position the mutation as close to the middle as possible. Only the reads that matched these sequences were taken into account in order to calculate the VAF of each of the triplicates. The possible noise generated by the PCR and sequencing process was controlled by eliminating the triplicates that exceeded the mean VAF + 1 standard deviation and by removing those biomarkers whose VAF was below the LOD (calculated as the mean VAF in healthy control donor samples + 3 standard deviation). The final MRD value was defined by the mutation with the highest VAF, as explained above. Statistical analyses were performed with SPSS version 22 (IBM, Inc., Chicago). The R2 method assessed the linear relationship between the different variables under study. Comparisons between the two groups were performed using the non-parametric Mann–Whitney U test. P-values of ≤0.05 were considered to be significant.

## Results

### High correlation between CTCs and cfDNA samples

Of the 24 patients, 20 were ultimately analyzed. The remaining four were removed from these studies due to the absence of paired samples or because there were not enough reads after sequencing.

From pathogenic or probably pathogenic variants detected at diagnosis, different follow-up markers were selected as MRD markers, and the most frequently affected genes were DNMT3A (30%), *RUNX1* (25%), *NPM1* (20%), and *ASXL1, TP53, IDH1, and JAK2 (10%*, **Supplementary Figure S1** *)*. Variants in CHIP-associated genes were excluded as biomarkers for MRD (28).

**Figure 1:**
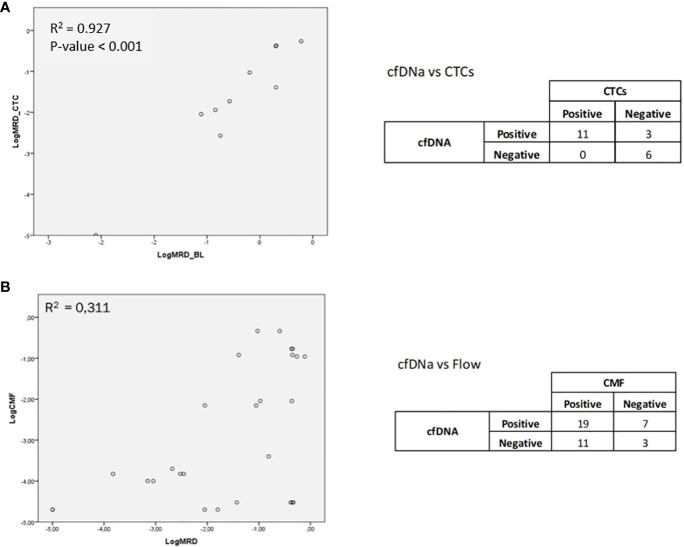
**(A)** Correlation between CTCs and cfDNA in paired samples. **(B)** Correlation between the MRD study by NGS and by MFC.

MRD assessment by cfDNA in the overall series showed MRD positivity in 13 out of 24 cases analyzed, being negative in 11 out of 24 cases. When studying the correlation between CTCs and cfDNA in paired samples, a good correlation was observed with an R^2^ value of 0.927 (p-value < 0.001) (**Figure 1A**).

### Correlations between MRD by cfDNA from Peripheral Blood and MRD by MFC from bone marrow

MFC data were obtained from 20 patients and compared with the results obtained by NGS in the MRD study. Of the 20 cases analyzed, MRD was detected by MFC in 15 cases and by NGS in 15 cases. It was observed that 12 of the 20 patients were coincident (positive or negative MRD detected by NGS and MFC) (**Supplementary Figure S2**), and 8 were discordant (positive MRD detected by NGS and negative MRD detected by MFC, or vice versa) (**Supplementary Figure S3**). The variants studied in the follow-up for the coincident cases were ETV6 (p.R418M), NPM1 (p.W288fs), RUNX1 (p.R162S), SF3B1 (p.K700E), DNMT3A (p.Arg882His), IDH1 (p.R132C), TP53 (p.R248Q), WT1 (p.K459fs), JAK2 (p.D319N), and PHF6 (p.R116*). We have monitored DNMT3A to verify that DNMT3A p.Arg882His should not be used as an MRD biomarker, although we have previously found other variants in DNMT3A with MRD utility.

The variants studied for the discordant cases were TP53 (p.C275Y), RUNX1 (p.R107C), TP53 (p.H193R), NRAS (p.E63K), NPM1 (p.W288fs), RUNX1 (p.W106R), IDH2 (p.R140Q), and RUNX1 (p.T214I).

In addition, four of the patients who were found to be MRD-positive by MFC were not detected by NGS, and four of the patients who were found to be MRD-positive by NGS were not detected by MFC. We detected a low correlation between the quantification of MRD by NGS and by MFC, with R2 = 0.311 (p-value = 0.016) (**Figure 1B**). In this case, it must be taken into consideration that two different tissues, BM and PB, and two different fractions, cells and free DNA, were being compared.

### Higher tumor signals in cfDNA than in CTC

**Figure 2:**
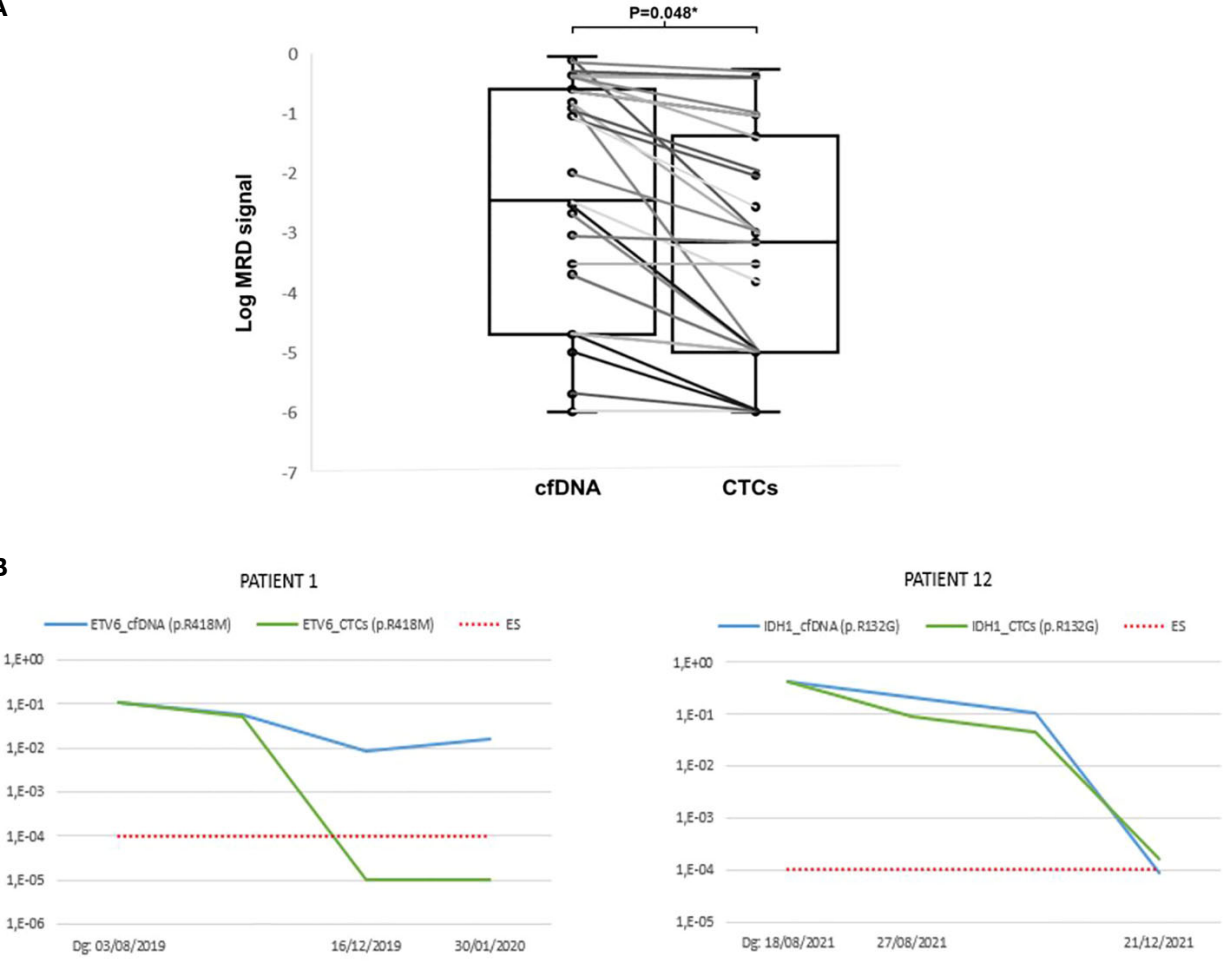
**(A)** MRD signal by NGS in cfDNA vs. CTCs. **(B)** Examples of patients where higher sensitivity was observed in cfDNA samples compared to CTCs samples. The X-axis represents the date on which the sample was obtained and the Y-axis represents the Variant Allele Frequency (VAF) of the sample.

In most cases, the signals observed in cfDNA were higher than in CTCs, with a median cfDNA of 0.0035 (range 0–0.89) vs. median CTCs of 0.0007 (range 0–0.55; P=0.048, **Figure 2A**). To demonstrate the applicability of the method, it was observed in some of the patients that cfDNA samples showed a higher sensitivity compared to CTC samples. In 13 of the 20 patients studied, the following statement is observed. In patient 1 (**Figure 2B**), monitoring of the variant p.R418M in the *ETV6* gene was studied. Disease detection was observed in the cfDNA sample but was no longer detected in the CTC sample. This patient relapsed and eventually died, so applying this method achieved greater sensitivity in the cfDNA sample. In patient 20 (**Figure 2B**), the variant p.R132G of the *IDH1* gene was studied. A very strong correlation between the two samples was observed, both of which ended up being negative. This patient was considered negative for MRD and did not relapse, although he eventually died some time later in complete remission.

### Challenges for the application of liquid biopsy to the study of MRD by NGS

#### *The* selection of a proper marker is essential for the LiqBio-MRD test

The MRD outcome was defined by the biomarker with the highest VAF, excluding variants of uncertain significance (VUS), missense and non-frameshift variants, and variants classified as non-eligible by Robert P. Hasserjian (28). These include clonal hematopoiesis variants (*DNMT3A, ASXL1, TET2, SRSF2*, and *BCOR*), myeloid neoplasia variables (*STAG2, JAK2, CALR*, and *MPL*), and those with variable significance (*IDH1, IDH2, RUNX1*, and *TP53*).

**Figure 3:**
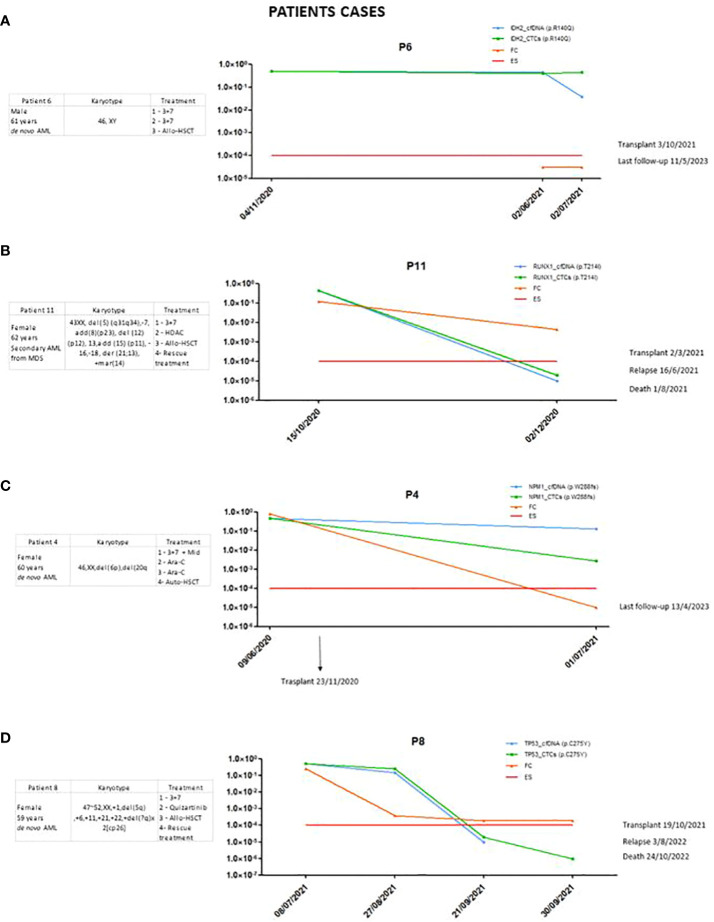
**(A)** Patient in whom the importance of choosing a good marker was shown. **(B)** Patient in whom the sensitivity of the marker was significantly reduced. **(C, D)** Patients in whom there was no clearance of the marker after treatment.

In the case of one patient (**Figure 3A**), disease was detected in the follow-up cfDNA and CTCs samples, but not by the MFC technique. The variant selected for follow-up was p.R140Q in the *IDH2* gene. This patient was in complete remission at the time of the study. However, the variant monitored in the follow-up was observed at around 50% both at diagnosis and at follow-up. This is a preleukemic variant, as described in the new ELN indications, and therefore not a good marker for follow-up.

Even if the presented method works, special attention should be paid to the type of markers that are chosen for the follow-up of the disease, as good follow-up markers are not obtained in all cases. These markers may often correspond to preleukemic variants or clonal hematopoiesis of indetermined potential (CHIP).

#### It is recommended to study several markers to monitor MRD

It was observed that in some cases, sensitivity to certain markers decreased. In patient 11 (**Figure 3B**), the p.T214I variant in *RUNX1* was studied. At the time of diagnosis, it was detected with higher sensitivity using our method, but at follow-up, two months later, it was no longer detected, and this patient was considered negative for MRD. This did not occur with the MFC technique, which showed a positive result for MRD. This patient eventually relapsed and died six months later. When studying the panel using NGS on the BM sample at the time of relapse, it was observed that the variant was still detected in about 45% of VAFs. The sensitivity obtained with this marker was reduced, and we therefore consider it important to follow up on as many markers as possible.

#### Minimum quantity of cfDNA required to apply LiqBio-MRD methodology

The limitations of cfDNA mainly derive from the limited amount of cfDNA used, which can sometimes be insufficient to obtain adequate sensitivity for assessing MRD.

A median cfDNA of 20.9 ng was obtained in the samples analyzed. The minimum amount of cfDNA necessary to carry out the sequencing process is 15 ng. Those samples that did not reach the minimum amount needed were not considered for analysis. In addition, the amount of genomic DNA contamination that these samples could contain was considered by analyzing the gDNA/cfDNA ratio using the bioanalyzer electropherogram. Samples with a ratio higher than one were also excluded. It was noted that the preservation of the sample and the timeframe in which it was processed were crucial.

#### cfDNA clearance and its correlation with clinical outcomes

In the cases of certain patients, we did not observe a good correlation between different techniques which are used to evaluate MRD, such as in patient 4 (**Figure 3C**), with NPM1 as the MRD biomarker. In the follow-up sample, MRD positivity was detected by our method post-transplant but was no longer detected by MFC. When *NPM1* was studied using qRT-PCR at the same point, it was not detected. The patient has not relapsed or died to date.

In patient 8 (**Figure 3D**), the p.C275Y variant in the *TP53* gene was studied. Notably, little clearance of the post-induction *TP53* variant was observed in this patient, who ultimately relapsed and died. However, in subsequent samples, no clearance was detected in cfDNA.

## Discussion

In this study, we present data regarding the usefulness of liquid biopsy as a marker of MRD in a cohort of 20 patients with AML. Herein, we have evaluated the role of plasma cell-free DNA in MRD monitoring and confirmed its usefulness, although some challenges remain. We have validated our NGS method with a sensitivity of 1/10,000 to detect MRD in the cfDNA of this cohort.

In recent years, there has been growing interest in the potential of cfDNA as a diagnostic and monitoring tool in various cancers, including AML. The release of fragmented DNA from cancer cells into the bloodstream can provide valuable information about the genetic mutations and alterations present in a tumor. However, it is important to note that while cfDNA analysis holds promise in AML, it is still a relatively new field of research, and its clinical utility is being actively investigated.

We observed a high concordance between CTC and cfDNA in this series of AML cases, unlike the frequent discordance observed in other liquid biopsy studies of solid tumors such as prostate cancer (29) or NSCLC (30), likely related to the type of tumor studied. AML is a type of cancer that affects the bone marrow and blood, leading to the abnormal production of immature white blood cells. Then, in leukemia, blastic or tumoral cells frequently circulate in the peripheral blood (CTCs), which does not occur in other tumors. Although tumor cell detachment is a phenomenon observed in several types of cancer, the specific features of AML contribute to the observed concordance between CTC and cfDNA in this particular leukemic context (31).

However, we observed a higher mutational burden in cfDNA than in CTCs in most analyzed cases of these AML patients. AML is known for its high genetic and clonal heterogeneity (32), meaning that different cancer cells within the same tumor can have distinct genetic alterations. CfDNA provides a more comprehensive representation of tumor heterogeneity because it contains DNA fragments from multiple tumor clones. In contrast, CTCs are individual cells and may not capture the full spectrum of genetic alterations present in a tumor. Therefore, the use of cfDNA may increase the sensitivity of peripheral blood for detection of MRD. Studies have shown that the use of extracellular vesicles obtained from PB samples can be used for the detection of prognostic and follow-up biomarkers in AML (33–38). In general, bone marrow was studied because it increases sensitivity by at least one logarithm versus peripheral blood for MRD evaluation in AML. However, numerous studies show discrepancies between the mutational profile of cfDNA and tumor biopsy (39–44). Clonal heterogeneity has even been described as a limitation of precision medicine (45).

We have observed that plasma cfDNA allows us to non-invasively obtain the data of tumor-related alterations through peripheral blood and capture tumor heterogeneity that may be missed in tissue biopsy, as has been previously described (46). Although we detected a significant percentage of cases in which the results coincided with the bone marrow MRD evaluation, there were discordant cases where the *TP53*-p.c275Y variant persisted with minimal clearance after induction therapy in cfDNA and CTCs, which does not correlate with the morphological response or MFC (flow cytometry). Indeed, the slow response and persistence of *TP53* variants in some cases may be indicative of treatment resistance. In certain instances, the presence of *TP53* variants that persist despite induction therapy can suggest a higher likelihood of treatment resistance or poor response to standard therapies. This resistance may be attributed to the specific genetic alterations and dysregulation of the *TP53* pathway, which plays a crucial role in cell cycle control and apoptosis (47, 48).

The main limitations of using cfDNA as an MRD marker are as follows: at least 20 ng of cfDNA is required, which is sometimes not achievable. The MagPurix^®^ cfDNA Extraction Kit LV (ZP02025) and EZ1&2 ccfDNA Kit (ref. 954854, Qiagen) were tested previously, however, the kit used for the isolation QIAamp Circulating Nucleic Acid Kit (ref. 55114, Qiagen) obtained the best quality and quantity of cfDNA. It is difficult to interpret the results, because not all markers detected at diagnosis are associated with transformation to AML and are more related to previous myelodysplastic syndromes (MDS) or clonal hematopoiesis; and there are still no data to define the cut-off point and the moment of evaluation after treatment with clinical significance. Another limitation that we have confirmed in this work is that more than one marker should be assessed (49); because we only used AML-related genes, some of them may have been associated with previous disorders, or have less sensitivity than expected from the technique.

### Utility of liquid biopsy

The molecular study of MRD using BM or CTC samples in peripheral blood has already been incorporated into clinical practice in certain subtypes of AML (e.g., CBF and NPM1). The advantage of studying cfDNA in the monitoring of patients with AML is that it provides a more representative view of bone marrow involvement (50) (than studying CTCs and has the additional benefit over bone marrow aspiration of being a non-invasive procedure. However, for the incorporation of these studies into clinical practice, standardization and clinical validation studies in these patients are necessary. The necessary process for these studies requires a procedure for designing primers specific to the patient’s mutational profile at diagnosis, optimizing the method to achieve a sensitivity of at least 10–4, and having a computational algorithm that allows for the rejection of amplification and sequencing errors.

### Correlation between CTCs and cfDNA

There are several factors that may influence the discrepancy in mutational burden observed between cfDNA and CTCs in patients with AML. Firstly, tumor clonal heterogeneity may contribute to differences in the release and circulation of tumor DNA in the blood. AML is known for its high genetic and clonal heterogeneity, which can result in the release of multiple tumor subclones into cfDNA, thereby increasing the detected mutational burden. In the other hand, cfDNA may originate from apoptotic or necrotic tumor cells, as well as circulating tumor cells, while CTCs represent viable tumor cells (51). This difference in sample origin and state may influence the quantity and quality of tumor DNA detected in cfDNA versus CTCs. This leads to greater or lesser differences depending on the timing of MRD monitoring. In the early stages, we observe more differences in mutational burden between cfDNA and CTCs, favoring cfDNA, likely related to the release of dead tumor cells after initial treatment. To mitigate the discrepancy and optimize the clinical utility of liquid biopsy in the context of AML, a deeper understanding of the underlying mechanisms driving the release and circulation of cfDNA and CTCs is required.

### Marker selection

Our marker selection was carried out following the recommendations of Robert P. Hasserjian (24) where clonal hematopoiesis variants (*DNMT3A, ASXL1, TET2, SRSF2*, and *BCOR*), myeloid neoplasia variants (*STAG2, JAK2, CALR*, and *MPL*), and those with variable significance (*IDH1, IDH2, RUNX1*, and *TP53*) were excluded as MRD marker. Other authors have published recommendations about MRD biomarker (28). However, in our experience, many variants detected in these genes are associated with the leukemia clone and serve as good MRD markers. The kinetics of each variant in response to treatments also provide us with information and help us define them as good MRD markers or not. This study has shown that it is important to understand and evaluate the kinetics of the variants being studied and not just to make decisions at a specific point. Studying the kinetics of mutations is a biological determinant of AML recurrence (52). In addition, a more detailed understanding of these kinetics would contribute to improved personalized decision making on the administration of a particular treatment (53). Artificial intelligence tools can assist us in monitoring this highly heterogeneous disease after generating follow-up data in cohorts of AML patients undergoing homogeneous treatments.

### Clinical translation and future directions

In addition to evaluating MRD in patients receiving intensive treatment, liquid biopsy could provide a non-invasive and dynamic assessment of treatment response over time and could be particularly beneficial in patients treated with targeted therapies or immunotherapy. However, further research is needed in prospective studies in larger patient cohorts and research efforts should focus on developing subtype-specific algorithms using machine learning tools.

## Conclusions

A method for the quantification of MRD by NGS was optimized using liquid biopsy techniques in acute myeloid leukemia. In addition, this method is applicable both when using CTCs (leukocyte DNA) and using cfDNA (circulating DNA in plasma). MRD quantification based on the use of cfDNA by NGS offers promising results and, in the future, could be a good option for disease monitoring or early detection of relapses in AML patients.

## Data availability statement

The data presented in the study are deposited in the SRA database, accession number PRJNA1120383.

## Ethics statement

The studies involving humans were approved by ethics committee of the Hospital Universitario 12 de Octubre. The studies were conducted in accordance with the local legislation and institutional requirements. The participants provided their written informed consent to participate in this study.

## Author contributions

NÁ performed research, interpreted data, and wrote the article. AM and SD conducted the bioinformatics analyses and interpreted data. LR and MR performed research. AG and LC provided samples. RC, RS, GC, IR, YH, and JM-L revised the article. SB designed and performed research, interpreted data, and wrote and revised the article. RA designed and performed research, interpreted data, and wrote, revised, and financed the article. All authors contributed to the article and approved the submitted version.

## References

[1] Welch JS Ley TJ Link DC Miller CA Larson DE Koboldt DC. The origin and evolution of mutations in acute myeloid leukemia. Cell. (2012) 150:264–78. doi: 10.1016/j.cell.2012.06.023 PMC3407563 22817890

[2] The Cancer Genome Atlas Research Network. Genomic and epigenomic landscapes of adult *de novo* acute myeloid leukemia. N Engl J Med. (2013) 368:2059–74. doi: 10.1056/NEJMoa1301689 PMC3767041 23634996

[3] Perl AE Altman JK Cortes J Smith C Litzow M Baer MR. Selective inhibition of FLT3 by gilteritinib in relapsed or refractory acute myeloid leukaemia: a multicentre, first-in-human, open-label, phase 1–2 study. Lancet Oncol. (2017) 18:1061–75. doi: 10.1016/S1470-2045(17)30416-3 PMC5572576 28645776

[4] Papaemmanuil E Gerstung M Bullinger L Gaidzik VI Paschka P Roberts ND. Genomic classification and prognosis in acute myeloid leukemia. N Engl J Med. (2016) 374:2209–21. doi: 10.1056/NEJMoa1516192 PMC4979995 27276561

[5] Leisch M Jansko B Zaborsky N Greil R Pleyer L. Next generation sequencing in AML—On the way to becoming a new standard for treatment initiation and/or modulation? Cancers. (2019) 11:252. doi: 10.3390/cancers11020252 30795628 PMC6406956

[6] Döhner H Wei AH Appelbaum FR Craddock C DiNardo CD Dombret H. Diagnosis and management of AML in adults: 2022 recommendations from an international expert panel on behalf of the ELN. Blood. (2022) 140:1345–77. doi: 10.1182/blood.2022016867 35797463

[7] Tiong IS Wei AH. New drugs creating new challenges in acute myeloid leukemia. Genes Chromosomes Cancer. (2019) 58:903–14. doi: 10.1002/gcc.22750 30861214

[8] Green SD Konig H. Treatment of acute myeloid leukemia in the era of genomics—Achievements and persisting challenges. Front Genet. (2020) 11:480. doi: 10.3389/fgene.2020.00480 32536937 PMC7267060

[9] Appelbaum FR Gundacker H Head DR Slovak ML Willman CL Godwin JE. Age and acute myeloid leukemia. Blood. (2006) 107:3481–5. doi: 10.1182/blood-2005-09-3724 PMC1895766 16455952

[10] Almeida AM Ramos F. Acute myeloid leukemia in the older adults. Leukemia Res Rep. (2016) 6:1–7. doi: 10.1016/j.lrr.2016.06.001 27408788 PMC4927655

[11] Schlenk RF Müller-Tidow C Benner A Kieser M. Relapsed/refractory acute myeloid leukemia: any progress? Curr Opin Oncol. (2017) 29:467–73. doi: 10.1097/CCO.0000000000000404 28857842

[12] Schuurhuis GJ Ossenkoppele GJ Kelder A Cloos J. Measurable residual disease in acute myeloid leukemia using flow cytometry: approaches for harmonization/standardization. Expert Rev Hematol. (2018) 11:921–35. doi: 10.1080/17474086.2018.1549479 30466339

[13] Terwijn M Van Putten WLJ Kelder A van der Velden VHJ Brooimans RA Pabst T. High prognostic impact of flow cytometric minimal residual disease detection in acute myeloid leukemia: data from the HOVON/SAKK AML 42A study. JCO. (2013) 31:3889–97. doi: 10.1200/JCO.2012.45.9628 24062400

[14] Jovanovic JV Ivey A Vannucchi AM Lippert E Oppliger Leibundgut E Cassinat B. Establishing optimal quantitative-polymerase chain reaction assays for routine diagnosis and tracking of minimal residual disease in JAK2-V617F-associated myeloproliferative neoplasms: a joint European LeukemiaNet/MPN&MPNr-EuroNet (COST action BM0902) study. Leukemia. (2013) 27:2032–9. doi: 10.1038/leu.2013.219 PMC3806250 23860450

[15] Gupta R Aggarwal G Rahman K Singh M Nityanand S. Acute myeloid leukemia following radioiodine therapy: Case report and brief literature review. Clin Cancer Investig J. (2016) 5:246. doi: 10.4103/2278-0513.182065

[16] Malagola M Bernardi S Polverelli N Russo D. Minimal residual disease monitoring in acute myeloid leukaemia: are we ready to move from bone marrow to peripheral blood? Br J Haematol. (2020) 190:135–6. doi: 10.1111/bjh.16579 32191348

[17] Stasik S Burkhard-Meier C Kramer M Middeke JM Oelschlaegel U Sockel K. Deep sequencing in CD34+ cells from peripheral blood enables sensitive detection of measurable residual disease in AML. Blood Adv. (2022) 6:3294–303. doi: 10.1182/bloodadvances.2021006233 PMC9198930 35320339

[18] Percival M-E Lai C Estey E Hourigan CS. Bone marrow evaluation for diagnosis and monitoring of acute myeloid leukemia. Blood Rev. (2017) 31:185–92. doi: 10.1016/j.blre.2017.01.003 PMC5513766 28190619

[19] Thakral D Gupta R Sahoo RK Verma P Kumar I Vashishtha S. Real-time molecular monitoring in acute myeloid leukemia with circulating tumor DNA. Front Cell Dev Biol. (2020) 8:604391. doi: 10.3389/fcell.2020.604391 33363162 PMC7759522

[20] Walter RB Appelbaum FR Estey EH Bernstein ID. Acute myeloid leukemia stem cells and CD33-targeted immunotherapy. Blood. (2012) 119:6198–208. doi: 10.1182/blood-2011-11-325050 PMC3383202 22286199

[21] Ganzel C Manola J Douer D Rowe JM Fernandez HF Paietta EM. Extramedullary disease in adult acute myeloid leukemia is common but lacks independent significance: analysis of patients in ECOG-ACRIN cancer research group trials, 1980–2008. JCO. (2016) 34:3544–53. doi: 10.1200/JCO.2016.67.5892 PMC5074349 27573652

[22] Solh M Solomon S Morris L Holland K Bashey A. Extramedullary acute myelogenous leukemia. Blood Rev. (2016) 30:333–9. doi: 10.1016/j.blre.2016.04.001 27094614

[23] Nakamura S Yokoyama K Shimizu E Yusa N Kondoh K Ogawa M. Prognostic impact of circulating tumor DNA status post–allogeneic hematopoietic stem cell transplantation in AML and MDS. Blood. (2019) 133:2682–95. doi: 10.1182/blood-2018-10-880690 30936070

[24] Short NJ Patel KP Albitar M Franquiz M Luthra R Kanagal-Shamanna R. Targeted next-generation sequencing of circulating cell-free DNA vs bone marrow in patients with acute myeloid leukemia. Blood Adv. (2020) 4:1670–7. doi: 10.1182/bloodadvances.2019001156 PMC7189293 32324887

[25] Onecha E Linares M Rapado I Ruiz-Heredia Y Martinez-Sanchez P Cedena T. A novel deep targeted sequencing method for minimal residual disease monitoring in acute myeloid leukemia. Haematologica. (2019) 104:288–96. doi: 10.3324/haematol.2018.194712 PMC6355493 30093399

[26] Onecha E Ruiz-Heredia Y Martínez-Cuadrón D Barragán E Martinez-Sanchez P Linares M. Improving the prediction of acute myeloid leukaemia outcomes by complementing mutational profiling with *ex vivo* chemosensitivity. Br J Haematol. (2020) 189:672–83. doi: 10.1111/bjh.16432 32068246

[27] Jiménez-Ubieto A Poza M Martin-Muñoz A Ruiz-Heredia Y Dorado S Figaredo G. Real-life disease monitoring in follicular lymphoma patients using liquid biopsy ultra-deep sequencing and PET/CT. Leukemia. (2023) 37:659–69. doi: 10.1038/s41375-022-01803-x 36596983

[28] Hasserjian RP Steensma DP Graubert TA Ebert BL. Clonal hematopoiesis and measurable residual disease assessment in acute myeloid leukemia. Blood. (2020) 135:1729–38. doi: 10.1182/blood.2019004770 PMC7225688 32232484

[29] Gupta S Hovelson DH Kemeny G Halabi S Foo W Anand M. Discordant and heterogeneous clinically relevant genomic alterations in circulating tumor cells vs plasma DNA from men with metastatic castration resistant prostate cancer. Genes Chromosomes Cancer. (2020) 59:225–39. doi: 10.1002/gcc.22824 31705765

[30] Ntzifa A Londra D Rampias T Kotsakis A Georgoulias V Lianidou E. DNA Methylation Analysis in Plasma Cell-Free DNA and Paired CTCs of NSCLC Patients before and after Osimertinib Treatment. Cancers. (2021) 13:5974. doi: 10.3390/cancers13235974 34885084 PMC8656722

[31] Rejniak KA. Circulating tumor cells: when a solid tumor meets a Fluid Microenvironment. In: Rejniak KA, editor. Systems biology of tumor microenvironment. advances in experimental medicine and biology. Springer International Publishing, Cham (2016). p. 93–106. doi: 10.1007/978-3-319-42023-3_5 PMC5113997 27739044

[32] Hirsch P Tang R Abermil N Flandrin P Moatti H Favale F. Precision and prognostic value of clone-specific minimal residual disease in acute myeloid leukemia. Haematologica. (2017) 102:1227–37. doi: 10.3324/haematol.2016.159681 PMC5566032 28302711

[33] Bernardi S Farina M Bosio K Di Lucanardo A Leoni A Re F. Feasibility of leukemia-derived exosome enrichment and co-isolated dsDNA sequencing in acute myeloid leukemia patients: A proof of concept for new leukemia biomarkers detection. Cancers. (2022) 14:4504. doi: 10.3390/cancers14184504 36139664 PMC9497185

[34] Kunz F Kontopoulou E Reinhardt K Soldierer M Strachan S Reinhardt D. Detection of AML-specific mutations in pediatric patient plasma using extracellular vesicle–derived RNA. Ann Hematol. (2019) 98:595–603. doi: 10.1007/s00277-019-03608-y 30673813

[35] Kontopoulou E Strachan S Reinhardt K Kunz F Walter C Walkenfort B. Evaluation of dsDNA from extracellular vesicles (EVs) in pediatric AML diagnostics. Ann Hematol. (2020) 99:459–75. doi: 10.1007/s00277-019-03866-w 31932899

[36] Bernardi S Zanaglio C Farina M Polverelli N Malagola M Russo D. dsDNA from extracellular vesicles (EVs) in adult AML. Ann Hematol. (2021) 100:1355–6. doi: 10.1007/s00277-020-04109-z PMC8043941 32474620

[37] Hornick NI Huan J Doron B Goloviznina NA Lapidus J Chang BH. Serum exosome microRNA as a minimally-invasive early biomarker of AML. Sci Rep. (2015) 5:11295. doi: 10.1038/srep11295 26067326 PMC4650871

[38] Miljkovic-Licina M Arraud N Zahra AD Ropraz P Matthes T. Quantification and phenotypic characterization of extracellular vesicles from patients with acute myeloid and B-cell lymphoblastic leukemia. Cancers. (2021) 14:56. doi: 10.3390/cancers14010056 35008226 PMC8750511

[39] Sun M-Y Lin F-Q Chen L-J Li H Lin W-Q Du H-Y. Targeted next-generation sequencing of circulating tumor DNA mutations among metastatic breast cancer patients. Curr Oncol. (2021) 28:2326–36. doi: 10.3390/curroncol28040214 PMC8293138 34202466

[40] Wei L Xie L Wang X Ma H Lv L Liu L. Circulating tumor DNA measurement provides reliable mutation detection in mice with human lung cancer xenografts. Lab Invest. (2018) 98:935–46. doi: 10.1038/s41374-018-0041-8 29497175

[41] Tu H-Y Li Y-S Bai X-Y Sun Y-L Zheng M-Y Ke E-E. Genetic profiling of cell-free DNA from pleural effusion in advanced lung cancer as a surrogate for tumor tissue and revealed additional clinical actionable targets. Clin Lung Cancer. (2022) 23:135–42. doi: 10.1016/j.cllc.2021.09.002 34645582

[42] Perakis SO Weber S Zhou Q Graf R Hojas S Riedl JM. Comparison of three commercial decision support platforms for matching of next-generation sequencing results with therapies in patients with cancer. ESMO Open. (2020) 5:e000872. doi: 10.1136/esmoopen-2020-000872 32967919 PMC7513637

[43] van der Leest P Ketelaar EM van Noesel CJM van den Broek D van Boerdonk RAA Deiman B. Dutch national round robin trial on plasma-derived circulating cell-free DNA extraction methods routinely used in clinical pathology for molecular tumor profiling. Clin Chem. (2022) 68:963–72. doi: 10.1093/clinchem/hvac069 35616097

[44] Xu T Kang X You X Dai L Tian D Yan W. Cross-platform comparison of four leading technologies for detecting *EGFR* mutations in circulating tumor DNA from non-small cell lung carcinoma patient plasma. Theranostics. (2017) 7:1437–46. doi: 10.7150/thno.16558 PMC5436504 28529628

[45] Clinton TN Chen Z Wise H Lenis AT Chavan S Donoghue MTA. Genomic heterogeneity as a barrier to precision oncology in urothelial cancer. Cell Rep. (2022) 41:111859. doi: 10.1016/j.celrep.2022.111859 36543146 PMC9882421

[46] Bettegowda C Sausen M Leary RJ Kinde I Wang Y Agrawal N. Detection of circulating tumor DNA in early- and late-stage human Malignancies. Sci Transl Med. (2014) 6. doi: 10.1126/scitranslmed.3007094 PMC4017867 24553385

[47] Nechiporuk T Kurtz SE Nikolova O Liu T Jones CL D’Alessandro A. The TP53 apoptotic network is a primary mediator of resistance to BCL2 inhibition in AML cells. Cancer Discovery. (2019) 9:910–25. doi: 10.1158/2159-8290.CD-19-0125 PMC6606338 31048320

[48] Bernard E Nannya Y Hasserjian RP Devlin SM Tuechler H Medina-Martinez JS. Implications of TP53 allelic state for genome stability, clinical presentation and outcomes in myelodysplastic syndromes. Nat Med. (2020) 26:1549–56. doi: 10.1038/s41591-020-1008-z PMC8381722 32747829

[49] Heuser M Freeman SD Ossenkoppele GJ Buccisano F Hourigan CS Ngai LL. 2021 Update on MRD in acute myeloid leukemia: a consensus document from the European LeukemiaNet MRD Working Party. Blood. (2021) 138:2753–67. doi: 10.1182/blood.2021013626 PMC8718623 34724563

[50] Yeh P Hunter T Sinha D Ftouni S Wallach E Jiang D. Circulating tumour DNA reflects treatment response and clonal evolution in chronic lymphocytic leukaemia. Nat Commun. (2017) 8:14756. doi: 10.1038/ncomms14756 28303898 PMC5357854

[51] Colmenares R Álvarez N Barrio S Martínez-López J Ayala R. The minimal residual disease using liquid biopsies in hematological Malignancies. Cancers. (2022) 14:1310. doi: 10.3390/cancers14051310 35267616 PMC8909350

[52] Rothenberg-Thurley M Amler S Goerlich D Köhnke T Konstandin NP Schneider S. Persistence of pre-leukemic clones during first remission and risk of relapse in acute myeloid leukemia. Leukemia. (2018) 32:1598–608. doi: 10.1038/s41375-018-0034-z PMC6035153 29472724

[53] Ommen HB Touzart A MacIntyre E Kern W Haferlach T Haferlach C. The kinetics of relapse in *DEK-NUP214* -positive acute myeloid leukemia patients. Eur J Haematol. (2015) 95:436–41. doi: 10.1111/ejh.12511 25605311

