# Supplementary material for: Detection of minimal residual disease in acute myeloid leukemia: evaluating utility and challenges
Source: Front Immunol. 2024 Jun 13;15:1252258. doi: 10.3389/fimmu.2024.1252258 (PMC11210172; doi:10.3389/fimmu.2024.1252258)

Supplementary Material

Liquid Biopsy-Based Detection of Minimal Residual Disease in Acute Myeloid Leukemia: Evaluating the Utility and Challenges

Noemí Álvarez[1,2], Alejandro Martín[2,3], Sara Dorado[3,4], Rafael Colmenares[1] , Laura Rufián[1,3], Margarita Rodríguez[1,3], Alicia Giménez[1], Laura Carneros[1], Ricardo Sanchez[1], Gonzalo Carreño-Tarragona[1], Inmaculada Rapado[1], Yanira Heredia[3], Joaquín Martínez-López[1,2,5,6], Santiago Barrio[1,2,3]† y Rosa Ayala[*1,2,5,6]†.

[*]Corresponding author: Rosa Ayala (rayala@ucm.es)

1 Supplementary Data

Supplementary Material should be uploaded separately on submission. Please include any supplementary data, figures and/or tables.

Supplementary material is not typeset so please ensure that all information is clearly presented, the appropriate caption is included in the file and not in the manuscript, and that the style conforms to the rest of the article.

2 Supplementary Figures and Tables

For more information on Supplementary Material and for details on the different file types accepted, please see here.

2.1 Supplementary Figures

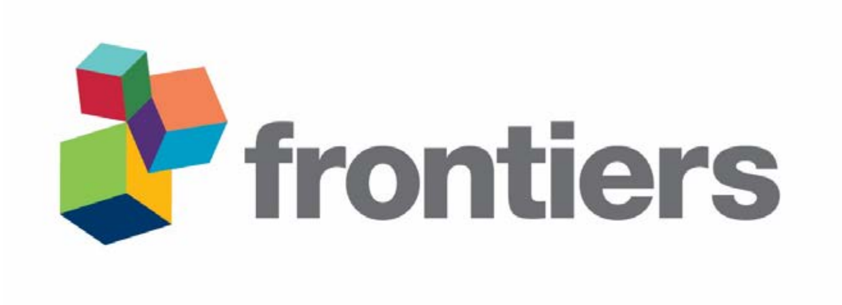

Supplementary Figure 1. The figure legends are required to have the same font as the main text, 12 point normal Times New Roman, single spaced. Please use a single paragraph for each legend and prepare the figures keeping in mind the PDF layout.

Supplemental Figure S1. Oncoplot showing the mutations of each patient at the time of diagnosis.

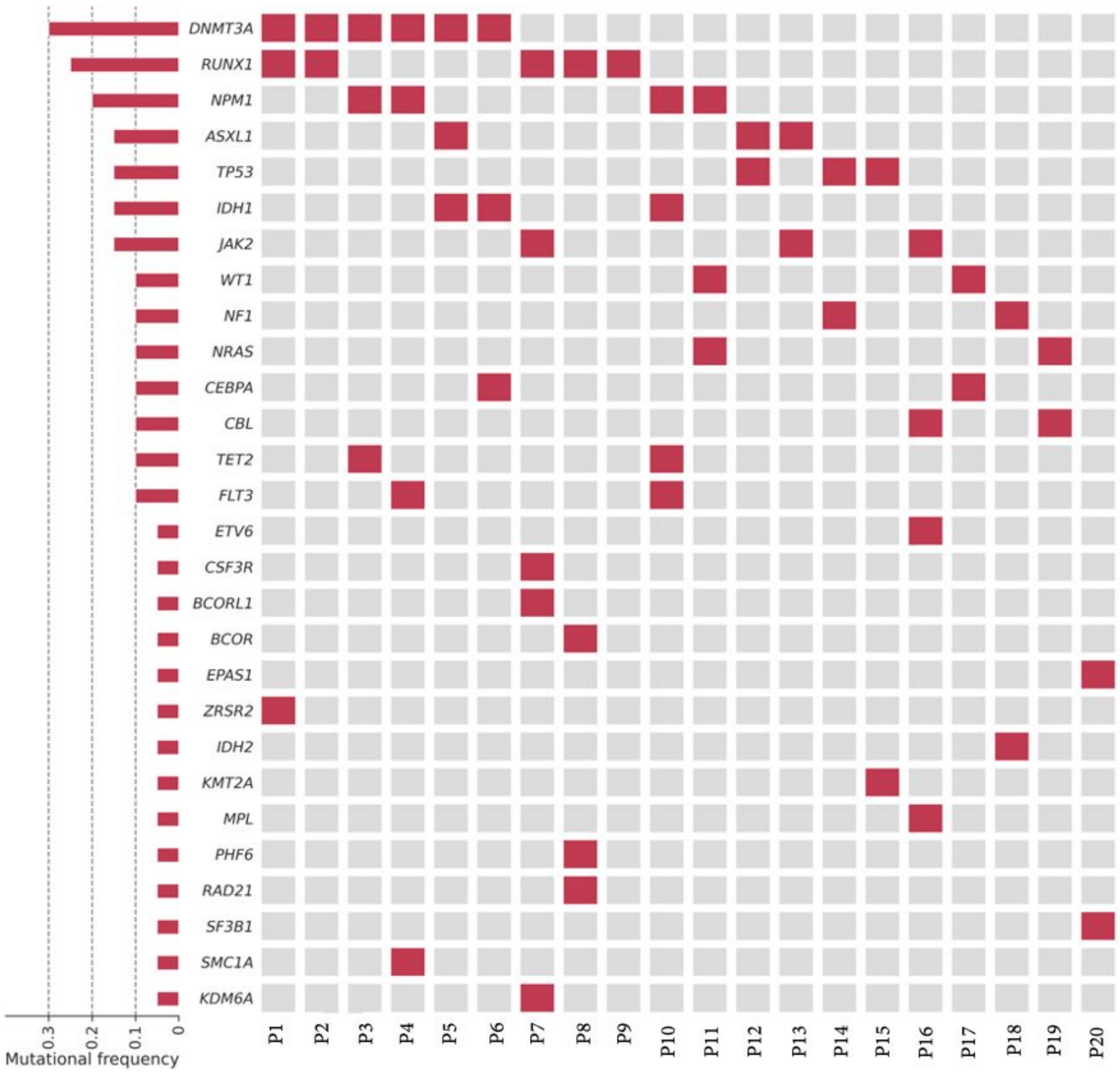

Supplemental Table S1. Clinical and biological characteristics of the patients.

Patient	Sex	Age	Type of AML	% Blasts at diagnosis	Leukocytes at diagnosis	ELN2022 classification	Karyotype	Treatment	Molecular characteristics
P1	Male	45	*De novo*	17	4.8	Favorable	46,XY	1 - Ida + AraC<br>2 - HDAC<br>3 - Allo-HSCT	CBL (7.4%), **ETV6** (10.6%), JAK2 (51.5%), MPL (50.5%)
P2	Female	72	*De novo*	11	9.1	Intermediate	46,XX	1 - 3+7<br>2 - 3+7 + Mid<br>3 - HDAC<br>4 - Auto-HSCT<br>5 - Aza<br>6 - Aza + Ven	DNMT3A (53.62%), FLT3 (35.86%), **NPM1** (40.48%), SMC1A (44.98%)
P3	Female	44	*De novo*	80	0.9	Favorable	46,XX[22]	1 - 3+7<br>2 - FLAG-IDA<br>3 - Aza + Ven	**NRAS** (3.53%), CBL (17.70%)
P4	Female	60	*De novo*	80	106.1	Intermediate	46,XX,del(6p),del(20q	1 - 3+7 + Mid<br>2 - Ara-C<br>3 - Ara-C<br>4- Auto-HSCT	FLT3 (2.80%), **NPM1** (47.10%), IDH1 (17.74%), TET2 (45.34%)
P5	Male	42	Secondary to RAEB	17	5.6	Intermediate	Clon1: 46,XY,t(7;19) (p12;q11)[3] ---- Clon2: 46,XY,del(5)(q31q33),t(7;19)(p12;q11)[13] ----Clon3: 46,XY,del(5)(q31q33),t(7;19)(p12;q11),del(20q)[7]	1 - IDA-FLAG<br>2 - Aza<br>3 -Quizartinib<br>4 - Ven<br>5 - Palliative treatment	**RUNX1** (7.49%)
P6	Male	61	*De novo*	-	3.3	Favorable	46, XY	1 - 3+7<br>2 - 3+7<br>3 - Allo-HSCT	**IDH2** (50.08%), NF1 (50.41%)
P7	Male	52	*De novo*	24	9.5	Favorable	46 XY	1 - 3+7<br>2 - 3+7<br>3 - Cita<br>4 - Allo-HSCT	**NPM1** (43.90%), NRAS (11.40%), WT1 (42.23%)
P8	Female	59	*De novo*	27	3.6	Adverse	47~52,XX,+1,del(5q) ,+6,+11,+21,+22,+del(?q)x2[cp26]	1 - 3+7 + Quizartinib (C.T.)<br>2 -3+7 + Quizartinib (C.T.)<br>3 - Allo-HSCT<br>4- Rescue treatment	ASXL1 (47.62%), **TP53** (52.02%)
P9	Female	45	*De novo*	28	3.5	Favorable	46 XX	1 -3+7 + Quizartinib (C.T.)<br>2 - 3+7 + Quizartinib (C.T.)<br>3 - Ara-C<br>4 - Allo-HSCT	CEBPA (34.43%), **WT1** (4.06%)
P10	Female	72	secondary to MM related to therapy	16	5.4	Adverse	45,XX,-7,t(9;22)(q34;q11) [3]/45,X,t(X;11)(q13;p15),-7,t(9;22)(q34;q11)[19]/45, X,del(X)(q?),-7,t(9;22)(q34;q11)[5]/46,XX,-7, t(9;22)(q34;q11)+mar[3]	1 - Aza<br>2 - Dasa<br>3 - Ven + Cita	**RUNX1** (36.75%), DNMT3A (41.97%), ZRSR2 (49.32%)
P11	Female	62	Secondary AML from MDS	14	2.6	Adverse	43XX, del(5) (q31q34),-7, add(8)(p23), del (12) (p12), 13,add (15) (p11), -16,-18, der (21;13), +mar(14)	1 - 3+7<br>2 - HDAC<br>3 - Allo-HSCT<br>4- Rescue treatment	**RUNX1** (45.91%), DNMT3A (6.82%)
P12	Female	42	*De novo*	50	3	Adverse	46,XX[30]	1 - 3+7<br>2 - 3+7<br>3 - 3+7<br>4 - Allo-HSCT	BCORL1 (9.20%). CSF3R (4.80%), JAK2 (50.20%), KDM6A (22.40%), **RUNX1** (36%)
P13	Female	64	*De novo*	-	4	Adverse	46,XX	1 - 3+7<br>2 - HDAC<br>3 - Allo-HSCT	**SF3B1** (43.11%), EPAS1 (49.71%)
P14	Male	41	Secondary to MDS	18	5.1	Adverse	trisomy 11 and t(X;17)	1 - Vyxeos<br>2 - Allo-HSCT	ASXL1 (26.6%), **JAK2** (49%)
P15	Female	47	*De novo*	13	1.9	Adverse	46,XX[20]	1 - 3+7<br>2 - Ara-C<br>3 - Allo-HSCT	BCOR (17.32%), **PHF6** (13.08%), RAD21 (7.89%), RUNX1 (11.44%)
P16	Female	73	*De novo*	77	84.3	Favorable	46,XX[30]	1 - 3+7 + Dauno<br>2 - Ara-C + Ida<br>3 - Cita<br>4 - Aza + Ven<br>5 - Aza	**NPM1** (34.87%), DNMT3A (39.89%), TET2 (9.77%)
P17	Female	55	*De novo*	46	18.7	Adverse	46,XX	1 - 3+7<br>2 - HDAC<br>3 - Allo-HSCT	IDH1 (43.21%), **DNMT3A** (45.52%), CEBPA (37.78%), STAG2 (3.45%)
P18	Female	84	*De novo*	38	0.8	Adverse	46,XX[28]	1 - Aza<br>2 - Palliative treatment	**IDH1** (29.4%), DNMT3A (41.2%), STAG2 (16.3%), ASXL1 (50%)
P19	Male	58	*De novo*	38	1.6	Adverse	43,Y,add(X)(p22.1),del(3) (q21),-5,-9,add (10)(q24),-13, add(17)(p12),-20,-22,+2mar [19]/46,XY[1]	1 - 3+7<br>2 - FLAG-IDA<br>3 - Aza + Ven	**TP53** (71.47%), KMT2A (50.46%)
P20	Female	66	*De novo*	44	18.39	Adverse	46,XX,del(5)(q13q33)[1] 41-42,XX,del(5)(q13q33),-7,dic(10;17)(q26;p13),-13,add(15)(p11),- 15,17,-18,der(22)t(22)(p11;p13)[cp4]	1 - Vyxeos<br>2 - FLAG-IDA	**TP53** (76.60%)

								3 - Allo-HSCT<br>4 - Aza	
P21	Male	45	*De novo*	70	6.1	Favorable	46,XY,t(7;11)(p15;p15)[18]/46,XY	1 - 3+7<br>2 - 3+7<br>3 - Ara-C<br>4 - Ara-C	RUNX1 (41.24%), ETV6 (38.94%)
P22	Male	59	*De novo*	80	44	Favorable	47,XY,+4[2]/46,XY[23]	1 - 3+7<br>2 - 3+7<br>3 - FLAG-IDA<br>4 - Ara-C<br>5 - Deci + Ven<br>6 - Allo-HSCT	NPM1 (42.7%), IDH2 (92.8%), RUNX1 (43.06%), DNMT3A (39.9%)
P23	Female	18	Secondary AML from MDS	-	4.4	Adverse	47,XX,+21c	1 - Deci<br>2 - Ara-C + Ida<br>3 - Ara-C + Ida<br>4 - Ara-C + Ida<br>5 - FLAG-IDA<br>6 - Deci + Ven<br>7 - Allo-HSCT	IDH1 (35.42%), SF3B1 (36.31%), ASXL1 (49.29%)
P24	Male	56	*De novo*	48	43.4	Adverse	46,XY[30]	1 - Vyxeos<br>2 - FLAG-IDA<br>3 - FLAG-IDA<br>4 - Allo-HSCT	ASXL1 (45.21%), SRSF2 (43.37%)

Supplemental Figure S2. MRD signal by NGS in cfDNA vs CTCs

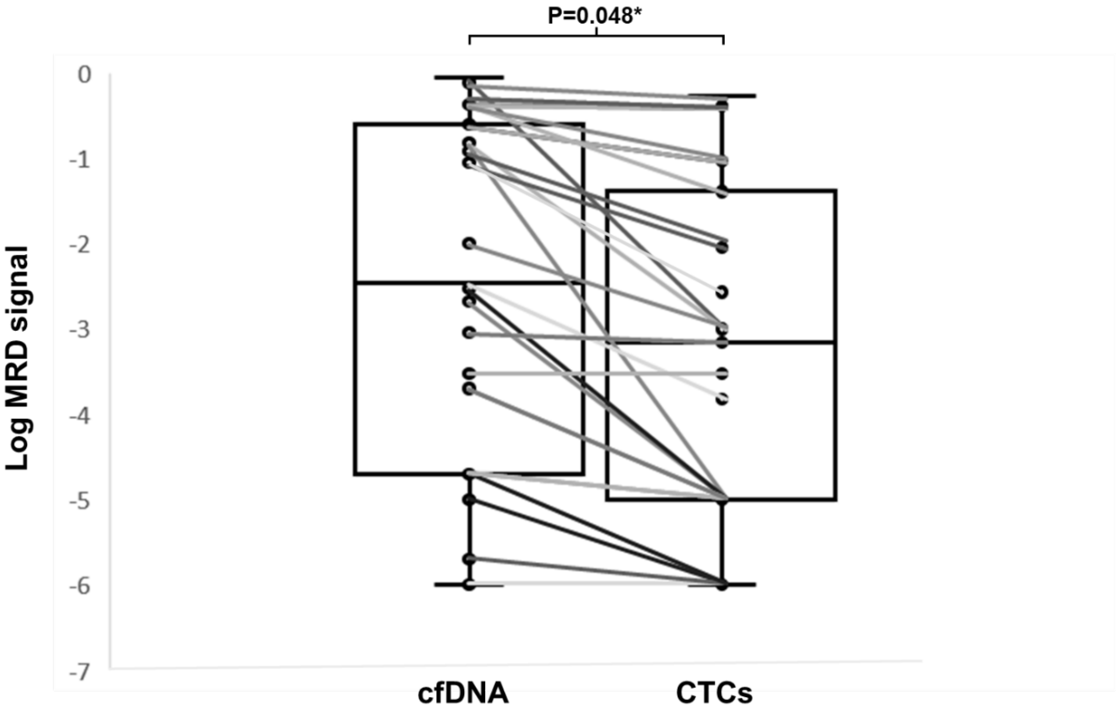

Supplemental Figure S3. Plots for all patients with coincident results obtained by the NGS and CBM techniques.

Patient 1	Karyotype	Treatment
Male<br>45 years<br>*de novo* AML	46,XY	1 - Ida + AraC<br>2 - HDAC<br>3 - Allo-HSCT

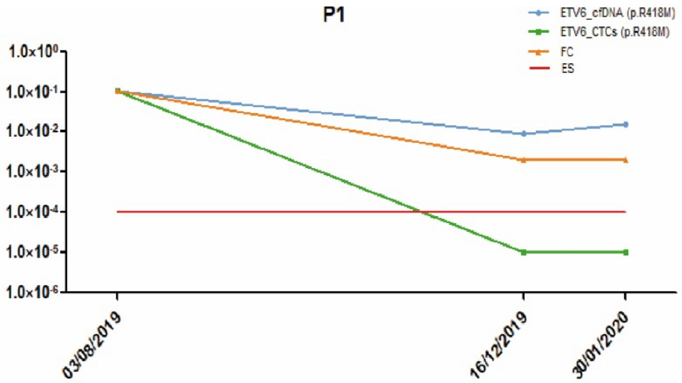

Patient 2	Karyotype	Treatment
Female<br>72 years<br>*de novo* AML	46,XX	1 - 3+7<br>2 - 3+7 + Mid<br>3 - HDAC<br>4 - Auto-HSCT<br>5 - Aza<br>6 - Aza + Ven

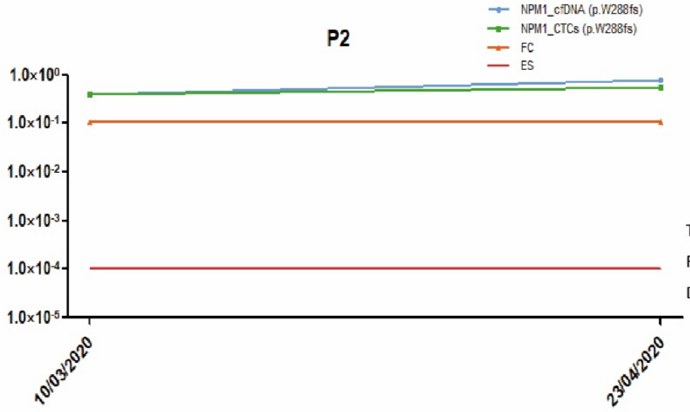

Patient 7	Karyotype	Treatment
Male<br>52 years<br>*de novo* AML	46 XY.	1 - 3+7<br>2 - 3+7<br>3 - Cita<br>4 - Allo-HSCT

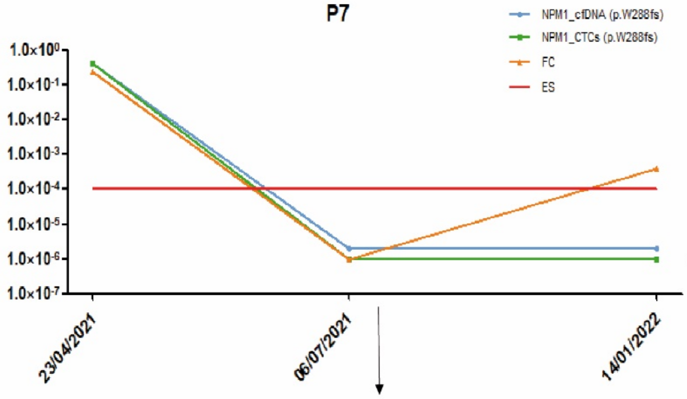

Patient 8	Karyotype	Treatment
Female<br>59 years<br>*de novo* AML	47~52,XX,+1,del(5q)<br>,+6,+11,+21,+22,+del(?q)x<br>2[cp26]	1 - 3+7<br>2 - Quizartinib<br>3 - Allo-HSCT<br>4- Rescue treatment

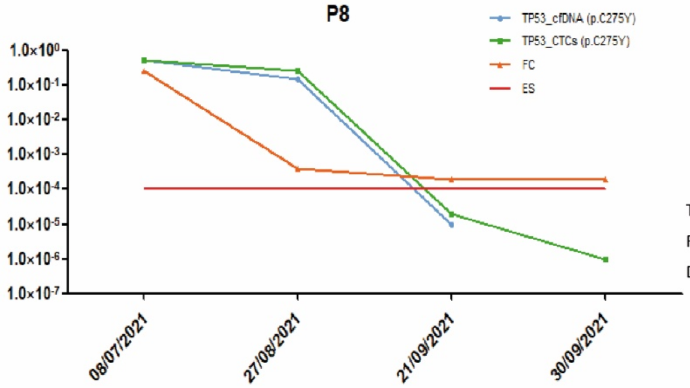

Patient 10	Karyotype	Treatment
Female<br>72 years<br>secondary to MM related to therapy	45,XX,-7,t(9;22)(q34;q11)[3]/45,X,t(X;11)(q13;p15),-7,t(9;22)(q34;q11)[19]/45,X,del(X)(q?),-7,t(9;22)(q34;q11)[5]/46,XX,-7,t(9;22)(q34;q11)+mar[3]	1 - Aza<br>2 - Dasa<br>3 - Ven + Cita

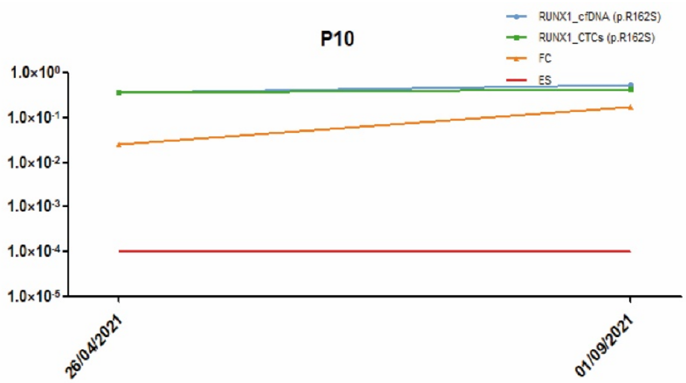

Relapse 25/10/2021

Death 05/05/2022

Patient 12	Karyotype	Treatment
Female<br>42 years<br>*de novo* AML	46,XX[30]	1 - 3+7<br>2 - 3+7<br>3 - 3+7<br>4 - Allo-HSCT

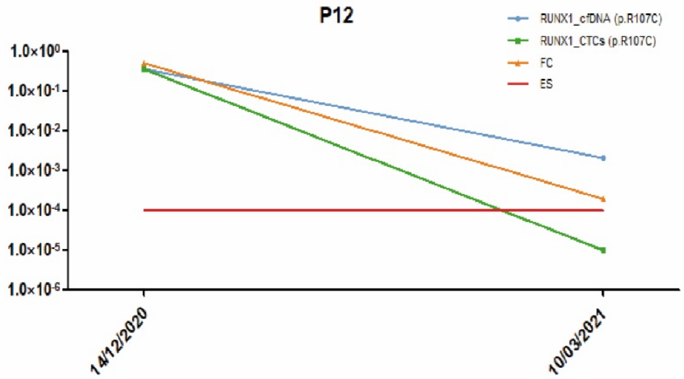

Transplant 24/5/2021

Last follow-up 23/03/2023

Patient 13	Karyotype	Treatment
Female<br>64 years<br>*de novo* AML	46,XX	1 - 3+7<br>2 - HDAC<br>3 - Allo-HSCT

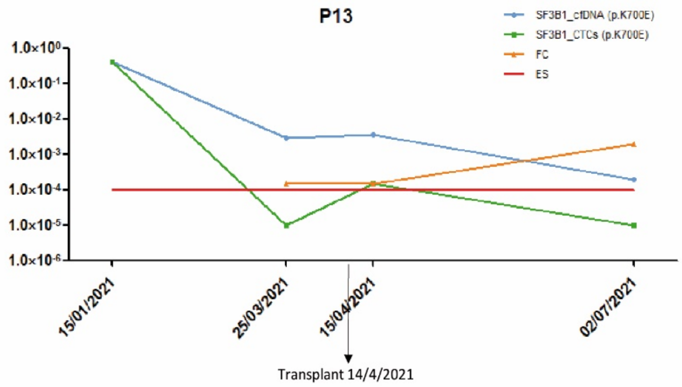

Relapse 10/11/2022

Last follow-up 17/04/2023

Patient 16	Karyotype	Treatment
Female<br>73 years<br>*de novo* AML	46,XX[30]	1 - 3+7 + Dauno<br>2 - Ara-C + Ida<br>3 - Cita<br>4 - Aza + Ven<br>5 - Aza

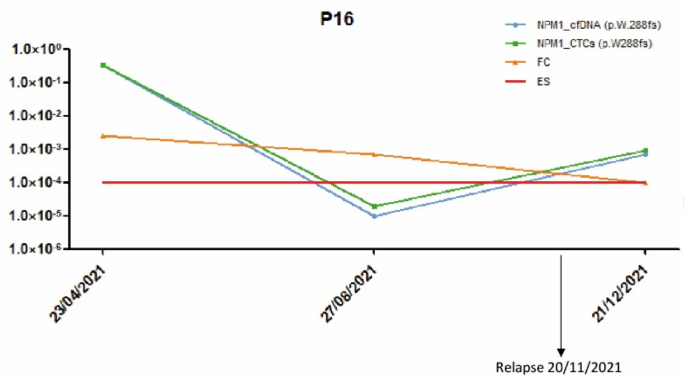

Death 30/01/2022

Patient 17	Karyotype	Treatment
Female<br>55 years<br>*de novo* AML	46,XX	1 - 3+7<br>2 - HDAC<br>3 - Allo-HSCT

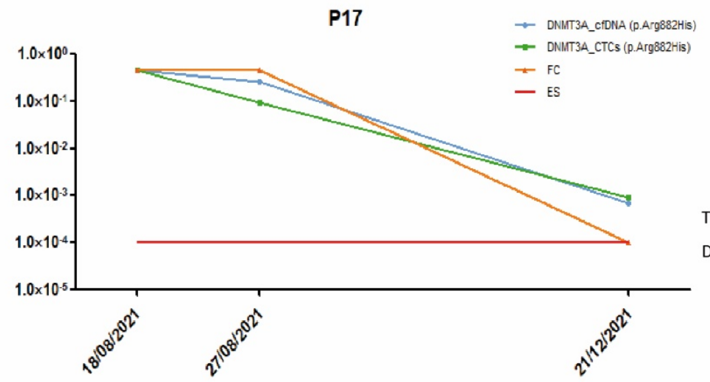

Transplant 30/3/2022

Death 13/6/2022

Patient 18	Karyotype	Treatment
Female<br>84 years<br>*de novo* AML	46,XX[28]	1 - Aza<br>2 - Palliative treatment

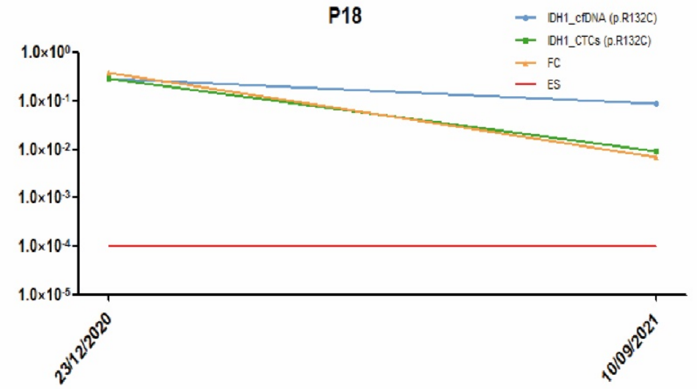

Death 15/3/2023

Patient 19	Karyotype	Treatment
Male<br>58 years<br>*de novo* AML	43,Y,add(X)(p22.1),del(3)(q21),-5,-9,add (10)(q24),-13, add(17)(p12),-20,-22,+2mar [19]/46,XY[1]	1 - 3+7<br>2 - FLAG-IDA<br>3 - Aza + Ven

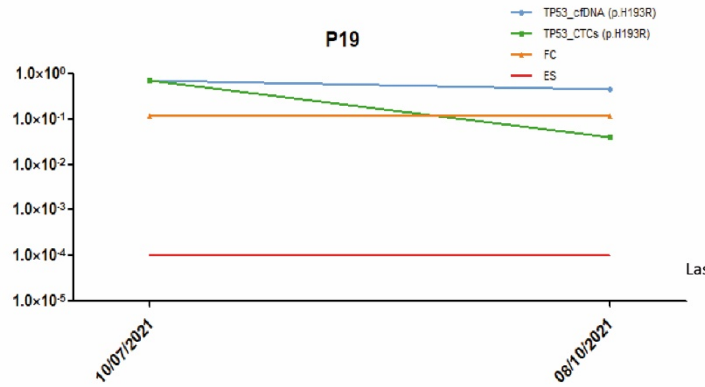

Last follow-up 26/10/2021

Patient 20	Karyotype	Treatment
Female<br>66 years<br>*de novo* AML	46,XX,del(5)(q13q33)[1] 41-42,XX,del(5)(q13q33),-7,dic(10;17)(q26;p13),-13,add(15)(p11),- 15,17,-18,der(22)t(22)(p11;p13)[cp4]	1 - Vyxeos<br>2 - FLAG-IDA<br>3 - Allo-HSCT<br>4 - Aza

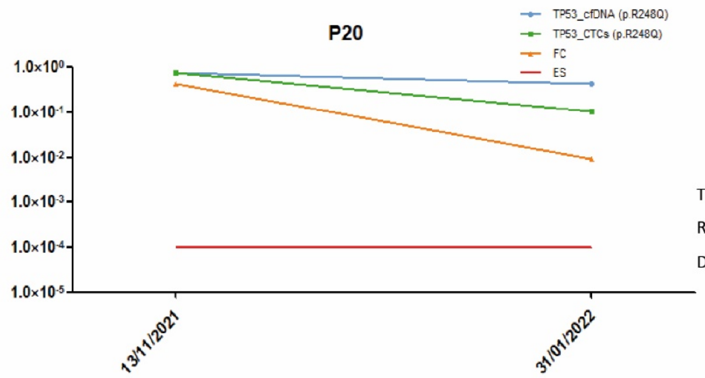

Transplant 05/07/2022

Relapse 22/08/2022

Death 17/03/2023

Supplemental Figure S4. Plots for all patients with non-coincident results obtained by the NGS and CBM techniques.

Patient 3	Karyotype	Treatment
Female<br>44 years<br>*de novo* AML	46,XX[22]	1 - 3+7<br>2 - FLAG-IDA<br>3 - Aza + Ven

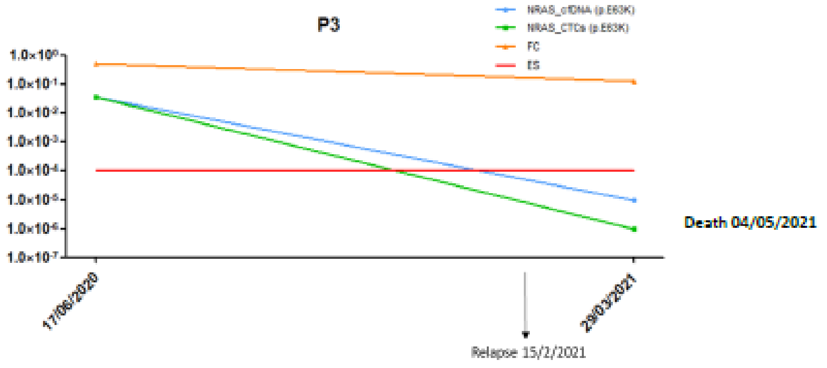

Patient 4	Karyotype	Treatment
Female<br>60 years<br>*de novo* AML	46,XX,del(6p),del(20q	1 - 3+7 + Mid<br>2 - Ara-C<br>3 - Ara-C<br>4- Auto-HSCT

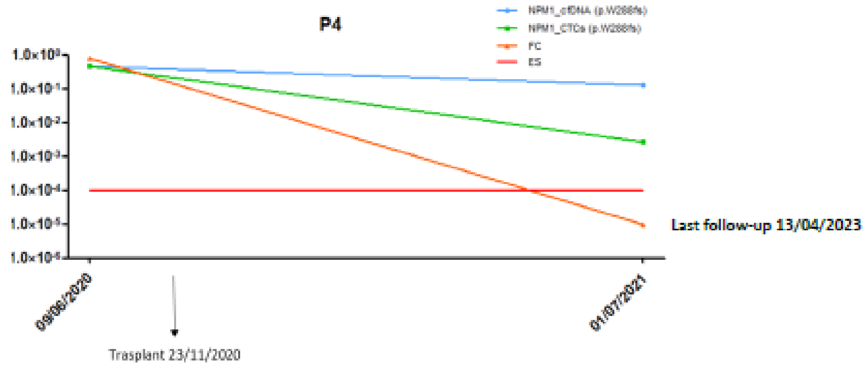

Patient 5	Karyotype	Treatment
Male<br>42 years<br>Secondary to<br>RAEB	Clon1: 46,XY,t(7;19)<br>(p12;q11)[3] ---- Clon2:<br>46,XY,del(5)(q31q33),t(7;1<br>9)(p12;q11)[13] ----Clon3:<br>46,XY,del(5)(q31q33),t(7;1<br>9)(p12;q11),del(20q)[7]	1 - IDA-FLAG<br>2 - Aza<br>3 - Quizartinib<br>4 - Ven<br>5 - Palliative<br>treatment

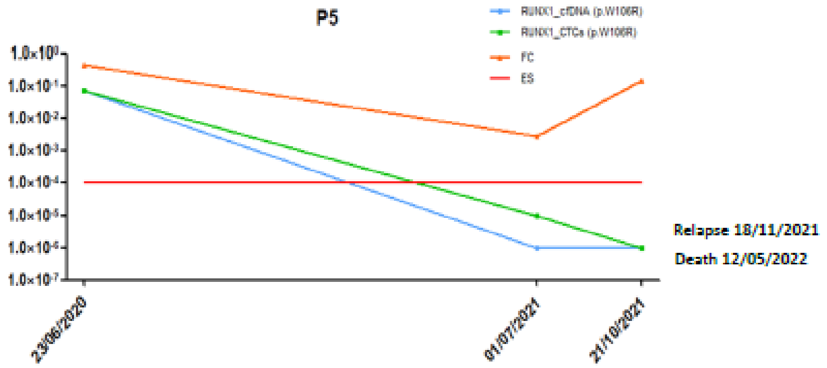

Patient 6	Karyotype	Treatment
Male<br>61 years<br>*de novo* AML	46, XY	1 - 3+7<br>2 - 3+7<br>3 - Allo-HSCT

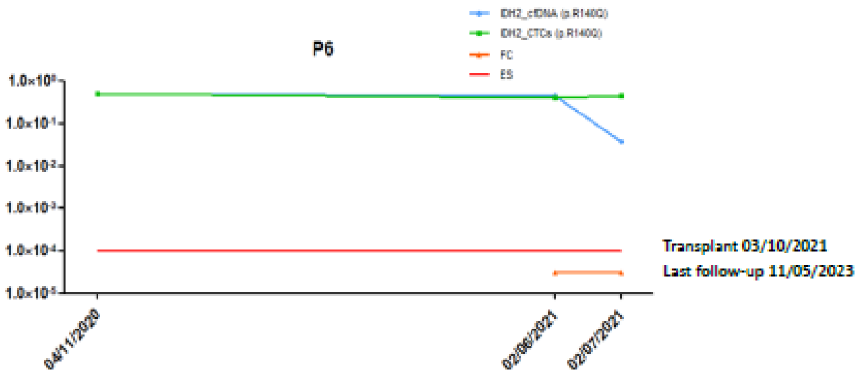

Patient 9	Karyotype	Treatment
Female<br>45 years<br>*de novo* AML	46 XX	1 - Quizartinib<br>2 - 3+7<br>3 - Ara-C<br>4 - Allo-HSCT

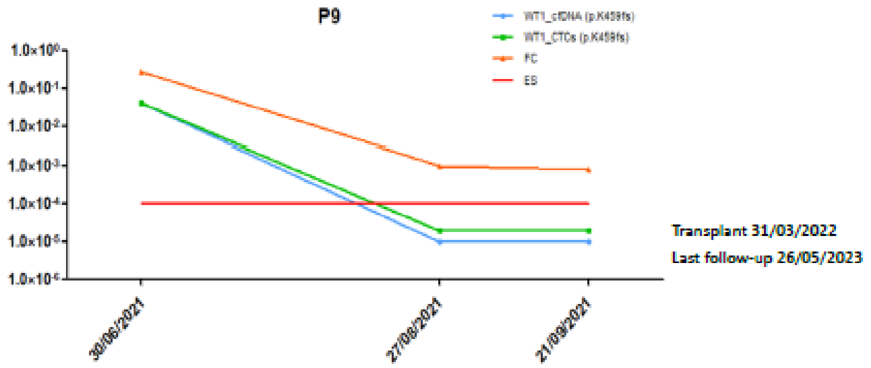

Patient 11	Karyotype	Treatment
Female<br>62 years<br>Secondary AML from MDS	43XX, del(5) (q31q34),-7, add(8)(p23), del (12) (p12), 13,add (15) (p11), -16,-18, der (21;13), +mar(14)	1 - 3+7<br>2 - HDAC<br>3 - Allo-HSCT<br>4- Rescue treatment

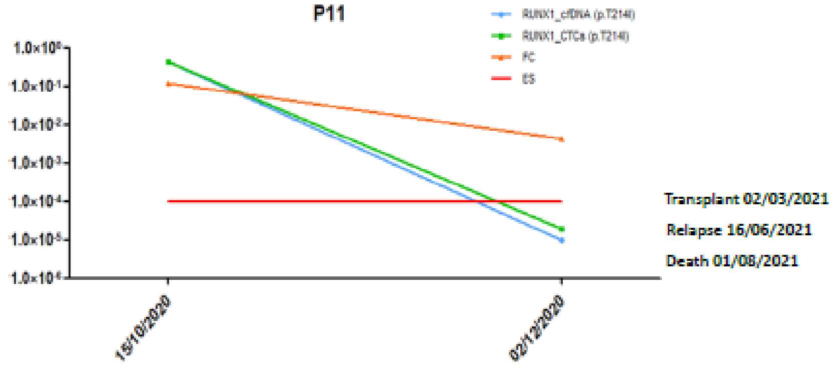

Patient 14	Karyotype	Treatment
Male<br>41 years<br>Secondary to MDS	trisomy 11 and t(X;17)	1 - Vyxeos<br>2 - Allo-HSCT

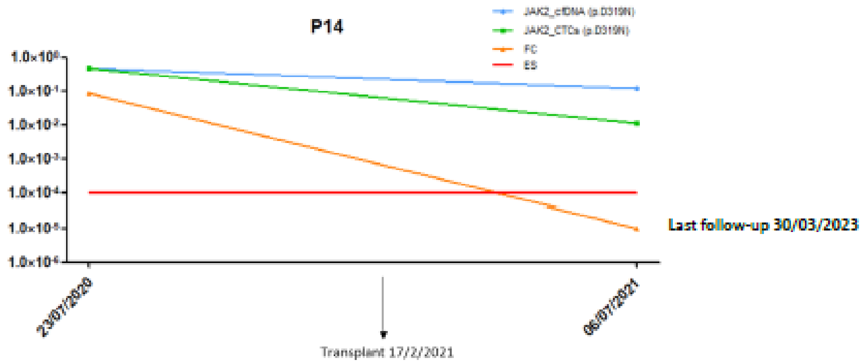

Patient 15	Karyotype	Treatment
Female<br>47 years<br>*de novo* AML	46,XX[20]	1 - 3+7<br>2 - Ara-C<br>3 - Allo-HSCT

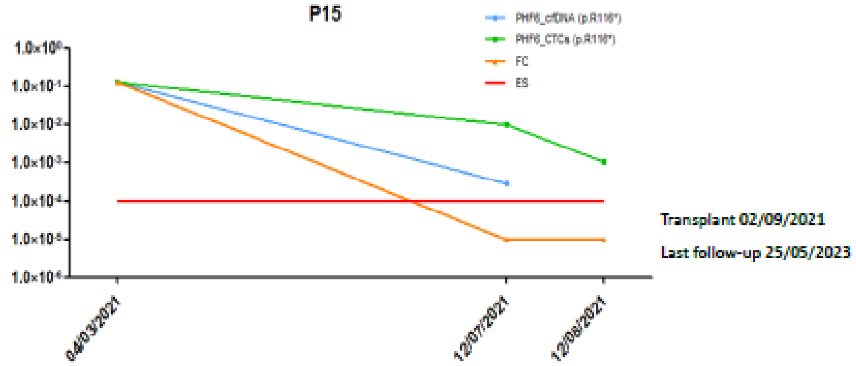

Supplement: Supplementary file 1 [file DataSheet_1.pdf]
